# Microfluidic Fabrication of Monodisperse and Recyclable TiO_2_-Poly(ethylene glycol) Diacrylate Hybrid Microgels for Removal of Methylene Blue from Aqueous Medium

**DOI:** 10.1021/acs.langmuir.3c02276

**Published:** 2023-12-14

**Authors:** Minjun Chen, Zahoor H. Farooqi, Guido Bolognesi, Goran T. Vladisavljević

**Affiliations:** † Department of Chemical Engineering, Loughborough University, Loughborough LE11 3TU, U.K.; ‡ School of Chemistry, University of the Punjab, New Campus, Lahore 54590, Pakistan; § Department of Chemistry, University College London, London WC1H 0AJ, U.K.

## Abstract

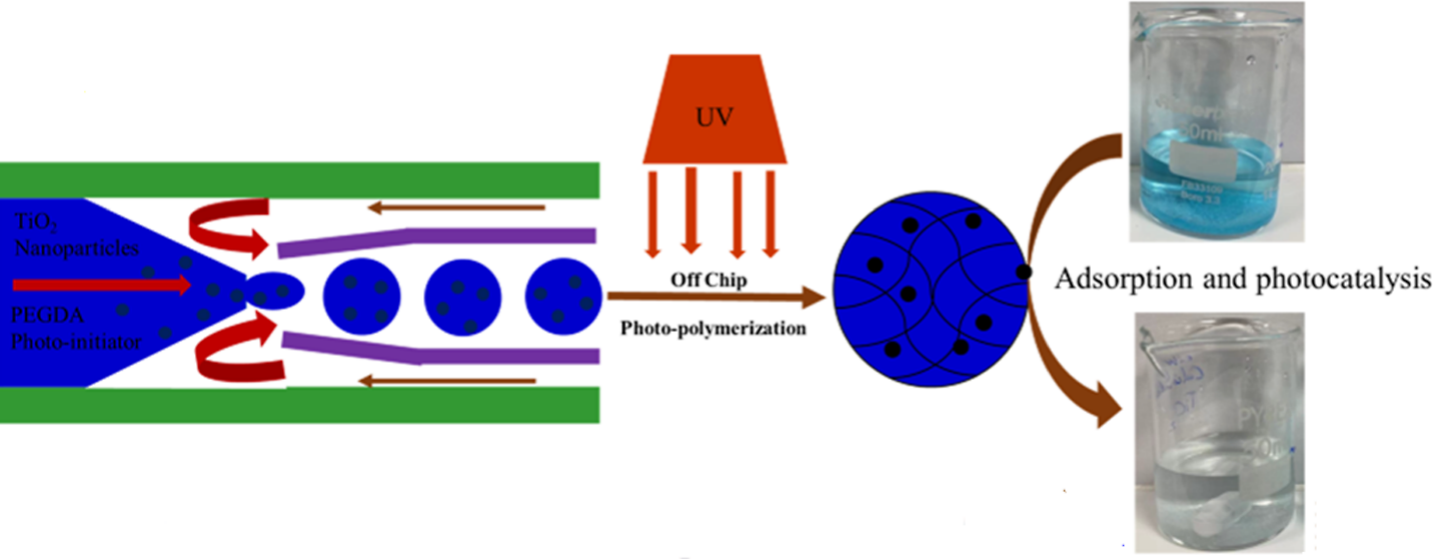

Nearly monodisperse titanium oxide–polyethylene glycol diacrylate [TiO_2_–P(EGDA)] hybrid microbeads containing 0.5 wt % TiO_2_ nanoparticles entrapped within a P(EGDA) cross-linked polymeric network were synthesized using a modular Lego-inspired glass capillary microfluidic device. TiO_2_–P(EGDA) hybrid microgels were characterized by optical microscopy, scanning electron microscopy, X-ray diffraction, energy dispersive X-ray spectroscopy, and thermogravimetric analysis. The fabricated TiO_2_–P(EGDA) hybrid microgel system showed 100% removal efficiency of methylene blue (MB) from its 1–3 ppm aqueous solutions after 4 h of UV light irradiation at 0.2 mW/cm^2^ at the loading of 25 g/L photocatalyst beads in the reaction mixture, corresponding to the loading of naked TiO_2_ of just 0.025 g/L. No decrease in photocatalytic efficiency was observed in 10 repeated runs with recycled photocatalyst using a fresh 1 ppm MB solution in each cycle. The rate of photocatalytic degradation was controlled by the UV light irradiance, catalyst loading, and the initial dye concentration. Physical adsorption of MB onto the surface of composite microgel was also observed. The adsorption data was best fitted with the Langmuir adsorption isotherm and the Elovich kinetic model. TiO_2_–P(EGDA) microgel beads are biocompatible, can be prepared with a tunable size in the microfluidic device, and can easily be separated from the reaction mixture by gravity settling. The TiO_2_–P(EGDA) system can be used for the removal of other toxic dyes and micropollutants from industrial wastewater.

## Introduction

Methylene blue (MB) is a widely used organic dye in industrial processes and can be found in large quantities in textile wastewater. It is highly poisonous and has toxic effects on humans and aquatic life.^1−3^ Therefore, various strategies have been developed to remove MB from aqueous solutions.^4,5^ However, adsorption and photocatalysis have gained the most attention as sustainable and eco-friendly technologies. Photocatalytic degradation of methylene blue has become a benchmark reaction to test the photocatalytic activity of organic–inorganic hybrid materials because the degradation can be easily monitored by a simple UV–visible spectrophotometric method. Polymeric microparticles and TiO_2_ nanoparticles (NPs) have been widely used as adsorbents and photocatalysts, respectively, for the removal of MB from wastewater.^6,7^ However, adsorption of MB onto polymeric particles is a slow process, and the adsorption capacity is often relatively small.^8^ On the other hand, the use of naked TiO_2_ NPs for photocatalytic degradation of MB is associated with some issues, such as the high aggregation (agglomeration) tendency of TiO_2_ NPs in aqueous solutions which results in reduced light absorbance and photocatalytic activity, and challenging separation from the reaction mixture after photocatalysis. Both problems can be addressed successfully by designing a hybrid material composed of polymeric microparticles and TiO_2_ NPs.^9−11^ Consequently, the use of inorganic–organic hybrid materials in adsorption, catalysis, and photocatalysis has become the subject of increasing interest.^12−20^ For example, Sun et al.^19^ developed an amino-functionalized TiO_2_ surface molecularly imprinted polymeric system using sodium lignosulfonate as a functional monomer for highly selective removal of MB via adsorption and photocatalysis methodologies. Idris et al.^21^ reported a TiO_2_/poly(vinyl alcohol)/cork hybrid material for scavenging of MB using adsorption and photocatalysis strategies. Wei et al.^22^ designed TiO_2_–P(EGDA) composite film using photopolymerization of P(EGDA) in the presence of TiO_2_ NPs and used the resulting film for adsorptive and photocatalytic removal of Congo red dye from aqueous medium. The loading of TiO_2_ NPs into polyethylene glycol diacrylate [P(EGDA)] hydrogel beads is advantageous because P(EGDA) is a hydrophilic, transparent, and biocompatible polymer that can entrap and immobilize TiO_2_ NPs while allowing UV light and reactants to pass through. In previously reported studies, TiO_2_ NPs have been incorporated only within bulk P(EGDA) hydrogel systems^23^ or P(EGDA) films.^22^ These studies encouraged us to load TiO_2_ NPs into P(EGDA) microbeads for adsorption and photocatalytic applications because micro hybrid beads are more efficient adsorbents and photocatalysts than bulk TiO_2_–P(EGDA) hybrid systems. To the best of our knowledge, fabrication of monodisperse TiO_2_-P(EGDA) hybrid microgels using Lego-inspired microfluidic device for adsorptive and photocatalytic removal of MB from water has not yet been reported in the literature. P(EGDA) microgel particles are highly monodisperse and big enough to be easily separated from water by gravity, with a separation efficiency of 100%. So, they can easily be recycled, and their mesh size is large enough for MB to diffuse inside but small enough to prevent loss of TiO_2_ NPs.

In the present work, we have used a novel Lego-inspired microfluidic device developed by our group to incorporate TiO_2_ NPs into P(EGDA) microgel for the first time. TiO_2_ NPs were dispersed in a prepolymer mixture, and the dispersion was emulsified by 3D microfluidic flow focusing, followed by off-chip photopolymerization of generated droplets. TiO_2_-P(EGDA) composite microgel particles have several advantages for adsorption and photocatalysis over bulk composite materials, including higher surface to volume ratio, high UV light transparency, and easy handling. Moreover, microgel particles can be efficiently brought into contact with the reaction mixture and used in various reactor designs, including fluidized bed reactors, packed bed reactors, and stirred tank reactors.

## Experimental Section

### Materials

P(EGDA) (*M*_w_ = 700 g/mol), titanium(IV) oxide (mixture of rutile and anatase, Degussa P25, <100 nm particle size (BET), purity 99.5%), and 2-hydroxy-40-(2-hydroxyethoxy)-2-methyl-propiophenone (Irgacure 2959) were purchased from Sigma-Aldrich, UK. MB was obtained from Fisher Bio-Reagents (Fisher Scientific, UK). XIAMETER PMX-200 Silicone Fluid, 100 cSt, and XIAMETER RSN-0749 Resin (50/50 mixture of trimethylsiloxysilicate and cyclomethicone) were received from Dow Chemical, USA. All chemicals were used as such without any further purification. Aqueous solutions were prepared using ultrapure water from a Millipore Milli-Q Plus 185 water purification system.

### Synthesis of TiO_2_–P(EGDA) Hybrid Microgels

**Figure 1:**
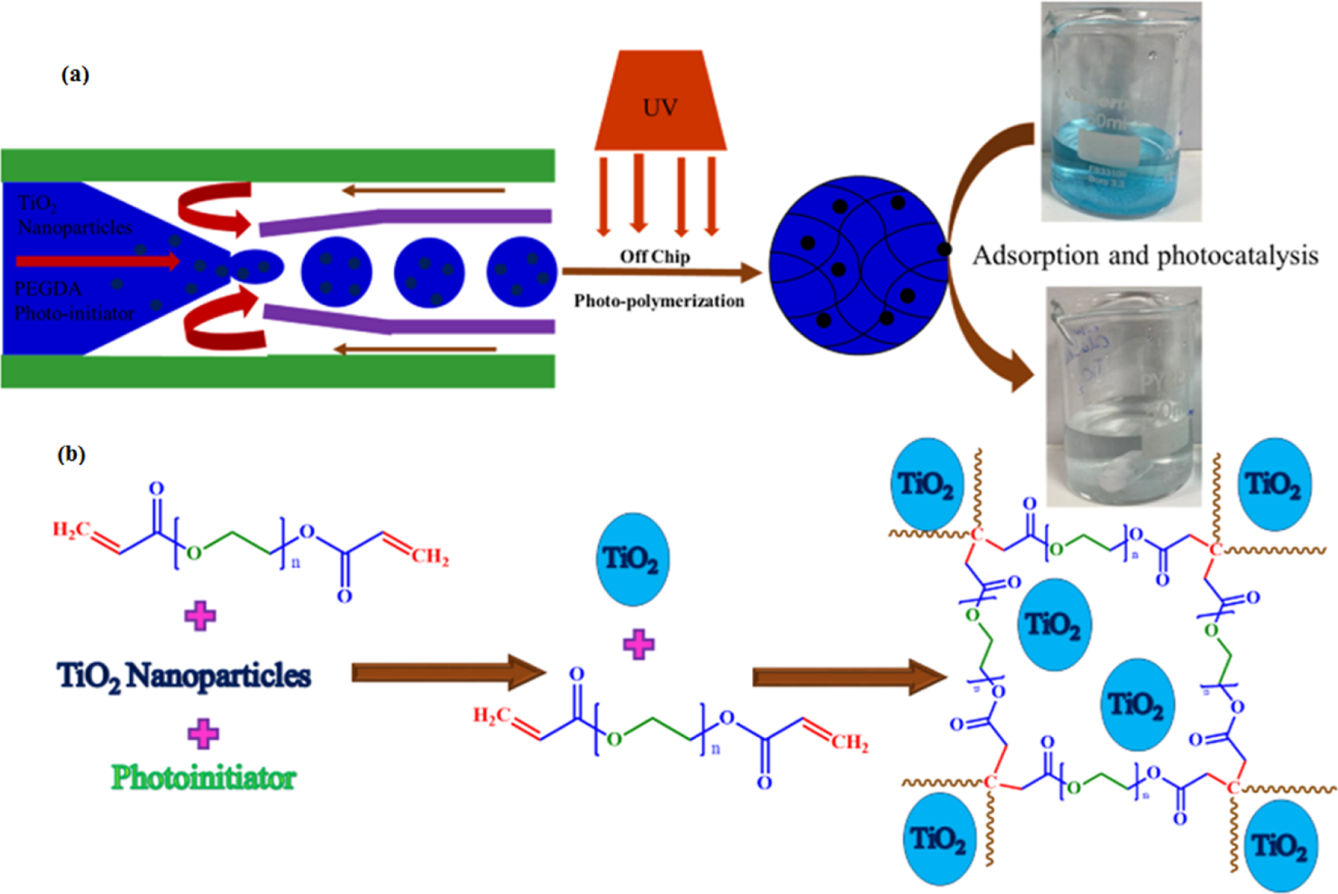
(a) Microfluidic fabrication of TiO_2_–P(EGDA) hybrid microbeads for the photocatalytic removal of methylene blue from aqueous solution. (b) Chemistry of photo-cross-linking of P(EGDA) in the presence of TiO_2_ NPs.

Water–oil (W/O) emulsion droplets containing TiO_2_ NPs, P(EGDA), and Irgacure 2959 were generated by 3D counter-current flow focusing in a two-phase Lego-inspired glass capillary device, as shown in Figure 1a.^24^ The schematic illustration for the photo-cross-linking of P(EGDA) in the presence of TiO_2_ NPs is given in Figure 1b. A full description of the microfluidic process and the experimental setup was provided in our previous work.^25^ Briefly, the dispersed phase was injected through an outer glass capillary into a tapered tip of the coaxially aligned inner capillary.

The dispersed phase jet was broken up into droplets due to jet instability induced by high flow velocity of the continuous phase at the tip of the inner capillary. The continuous phase was supplied countercurrently through the annular gap between the two capillaries. The device was placed on the stage of an inverted biological microscope, which served to control the microfluidic process.

The continuous phase was silicone oil (XIAMETER PMX-200) containing 3.5 wt % RSN-0749 surfactant and the dispersed phase was an aqueous solution containing 98.5 wt % P(EGDA) prepolymer, 1 wt % Igracure 2959, and 0.5 wt % TiO_2_. Pure P(EGDA) beads were prepared using the dispersed phase containing 99 wt % P(EGDA) prepolymer and 1 wt % Igracure 2959. The flow rate of the continuous phase and the dispersed phase was adjusted to 0.8 and 0.16 mL/h, respectively, using Harvard Apparatus model 11 Elite syringe pumps (Biochrom Ltd., UK). Both liquids were delivered from SGE gastight glass syringes (10 mL, Sigma-Aldrich, UK) connected to the device via polyethylene tubing (I.D. 0.86 mm, O.D. 1.52 mm, Smiths Medicals, Luton, UK). The tubing for the delivery of the dispersed phase was covered by aluminum foil to avoid premature polymerization of P(EGDA) by ambient light. The produced droplets were collected in a Petri dish prefilled with the continuous phase and exposed to UVA light (365–395 nm) for 6.5 min from a UVAHAND 250GS lamp (Dr. Hönle AG, Gilching, Germany) to cure the droplets. The UV light irradiance was 75 mW/cm^2^, as measured by a radiometer (365 nm, VLX-3W). The beads were washed 5–7 times with acetone and then several times with deionized water to fully remove the oil phase and unreacted species.

### Characterization of TiO_2_–P(EGDA) Hybrid Beads

Bright-field microscopy images of W/O emulsion droplets and TiO_2_–P(EGDA) hybrid beads were captured by using a CCD camera (Retiga 6000, Canada) interfaced to a computer running Q-capture software. The surface morphology of TiO_2_–P(EGDA) hybrid beads was examined by a field emission scanning electron microscope (JEOL, JSM 7800F, Tokyo, Japan) with backscatter (BED-C) detector mode operating at an acceleration voltage of 5.0 kV. X-ray diffraction (XRD) analysis was performed by using a D2 PHASER XRD diffractometer (Bruker UK Limited, UK). The TGA 550 instrument (TA Instruments, USA) was used to monitor the weight loss of dried particles of pure P(EGDA) microgel and TiO_2_–P(EGDA) hybrid microgel (12 mg each) under a nitrogen atmosphere in the temperature range of 20–600 °C at a rate of 10 °C/min.

### Photocatalytic Degradation of MB

In photocatalytic experiments, 0.13 g/L hybrid beads were stirred in 20 mL of MB solution (1 or 3 ppm), while the suspension was exposed to UV light at 2, 4, or 8 W/m^2^. In the experiments performed using different catalyst loadings, 100–500 mg of hybrid beads were added into 10 mL of 10 ppm MB solution, and the mixture was irradiated by UV light at 8 W/m^2^. In all experiments, the solution pH was kept at 7, and the residual MB concentration in the liquid phase was measured using a UV–visible spectrophotometer (NanoDrop One/OneC, Thermo Fisher Scientific, USA). The absorption spectra were acquired in the wavelength range 350–850 nm. In the adsorption experiments, 55 mg of hybrid beads were added into 30 mL of the dye solution containing between 0.25 and 7.5 ppm of MB at pH 7, and the mixture was stirred in the dark for a set amount of time. Adsorption isotherms were determined by stirring the suspension for 48 h and measuring the equilibrium MB concentration in the liquid phase. The adsorption kinetics was investigated by monitoring a decrease in the MB concentration with time in the first 18 min of the adsorption process. The control experiments were carried out under similar conditions.

## Results and Discussion

### Fabrication and Characterization of TiO_2_–P(EGDA) Hybrid Beads

**Figure 2:**
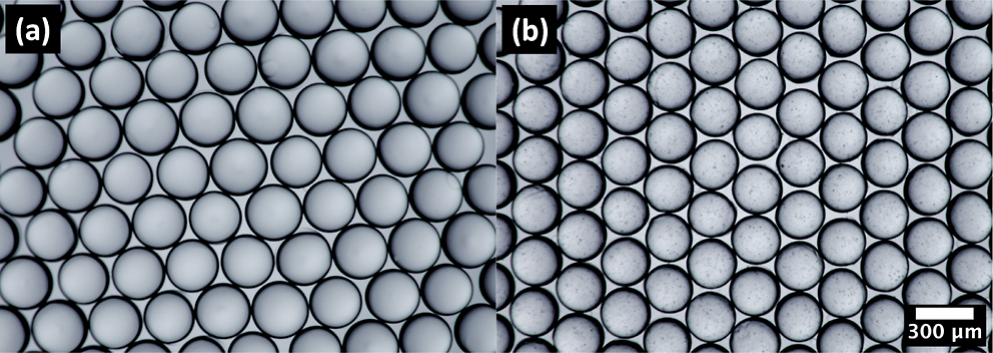
Optical microscopic images of (a) pure P(EGDA) beads and (b) TiO_2_–P(EGDA) hybrid beads. The same scale bar applies to both images.

Microscopic images of P(EGDA) beads and TiO_2_–P(EGDA) hybrid beads are shown in Figure 2a,b respectively. Both P(EGDA) beads and TiO_2_–P(EGDA) hybrid beads are nearly monodisperse, as can be seen by their hexagonal arrangement on a microscope slide, and have a spherical shape without any fused particles.

The size of the TiO_2_–P(EGDA) hybrid beads was slightly less than that of the P(EGDA) beads. TiO_2_ NPs embedded in the polymer matrix act as a physical cross-linker to cause shrinkage of the polymeric network. The average diameter of TiO_2_–P(EGDA) hybrid beads and pure P(EGDA) beads was 238 and 243 μm, respectively. Small dots uniformly distributed within the hybrid beads in Figure 2b indicate the presence of embedded TiO_2_ NPs.

**Figure 3:**
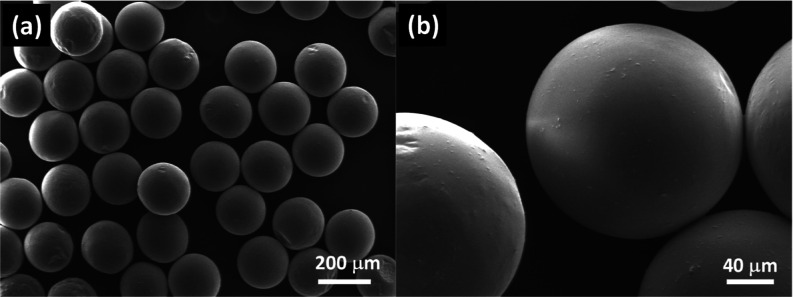
SEM images of TiO_2_–P(EGDA) beads at different magnifications, indicating their size, shape, and surface morphology. The scale bar is 100 μm in (a) and 10 μm in (b).

The morphological investigations of TiO_2_–P(EGDA) hybrid beads were carried out by SEM. The SEM images of TiO_2_–P(EGDA) hybrid beads at two different magnifications are shown in Figure 3a,b. TiO_2_–P(EGDA) hybrid beads shown in Figure 3b have a spherical shape with occasional wrinkles on the surface. TiO_2_ NPs are visible in Figure 3b in the form of dot-like protrusions scattered on the particle surface. Surface wrinkles on the microgel surface occur due to nonuniform droplet polymerization as a result of inhibition of free radicals at the droplet surface by oxygen dissolved in silicone oil. Nonpolymerized PEGDA molecules at the particle surface cause swelling of the cross-linked polymer in the interior of the particle, resulting in stresses that lead to surface buckling and wrinkles.^26^

**Figure 4:**
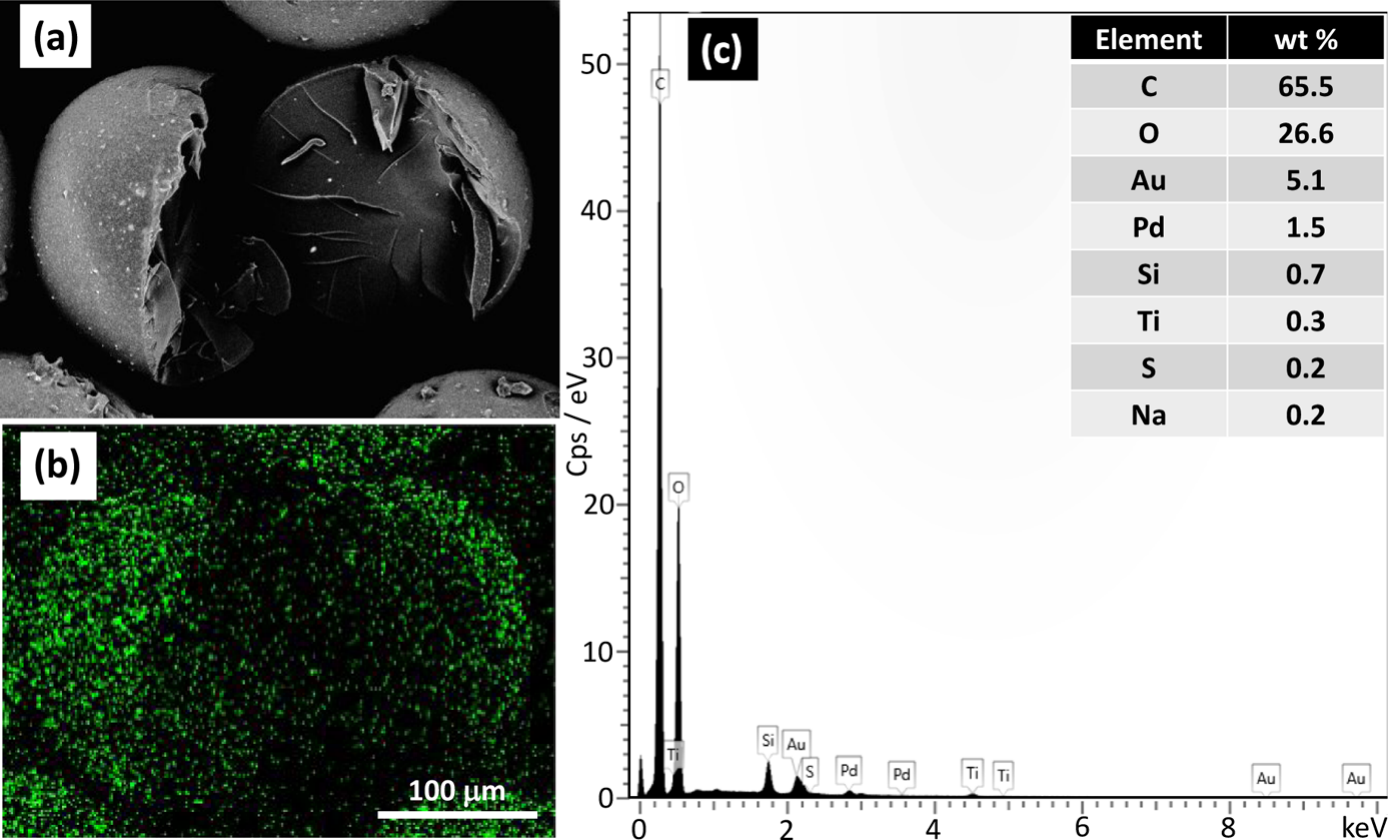
(a) SEM image of a single TiO_2_–P(EGDA) bead cut with a knife to expose TiO_2_ NPs inside the bead; (b) EDX mapping of titanium distribution within the bead; (c) EDX spectrum of a single hybrid bead.

Moreover, SEM, in combination with energy dispersive X-ray spectroscopy (EDX), was used to investigate the distribution of TiO_2_ particles within hybrid beads. For this purpose, a single TiO_2_–P(EGDA) bead was cut into two pieces, and its SEM analysis with EDX mapping was performed. The SEM image of the cut bead is shown in Figure 4a. Bright dots at the particle surface and in the cross section confirm the presence of TiO_2_ NPs both in the interior of the beads and on the surface. TiO_2_ NPs in the interior of the beads should be accessible to the dye molecules since MB molecules have a rectangular cross section with a surface area of 0.76 × 0.33 nm,^27^ and the mesh size of P(EGDA) polymer network in aqueous medium at pH 7 is about 1.1 nm, as calculated based on the end-to-end distance of PEGDA prepolymer chains and the swollen polymer volume fraction.

The EDX elemental mapping in Figure 4b shows that titanium is distributed in all regions on the surface and in the interior of the beads. Green dots of different sizes and shapes suggest that TiO_2_ NPs are partially aggregated, which is expected since no dispersant was used to stabilize TiO_2_ NPs. The agglomeration of TiO_2_ NPs can be reduced by adding a water-soluble surfactant^28^ or by coating TiO_2_ NPs with polymers such as polyethylene glycol (PEG), polyacrylamide (PAM), and poly(acrylic acid) (PAA) for steric stabilization of TiO_2_ sols.^29^

The EDX spectrum of the TiO_2_–P(EGDA) particle shown in Figure 4c reveals the presence of titanium (Ti) on the bead surface in addition to carbon (C) from the polymer backbone, oxygen (O) from TiO_2_ NPs and oxygen-containing groups of P(EGDA) (carbonyl and ether), gold (Au), and palladium (Pd) from the sputter coating process, and silicon (Si) from the traces of silicon-based oil and surfactant remaining on the particle surface after washing. Other elements present in small quantities, such as sulfur and sodium, are impurities from the raw materials.

**Figure 5:**
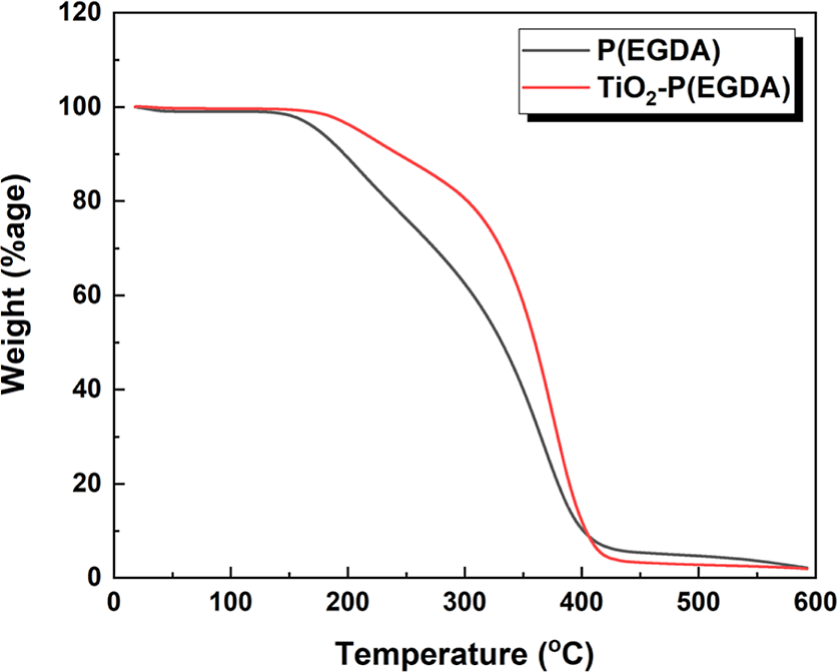
TGA curves of pure P(EGDA) and TiO_2_–P(EGDA) hybrid beads.

TGA analysis of P(EGDA) and TiO_2_–P(EGDA) beads was performed to determine their thermal stability and operating temperature ranges for photocatalysis and regeneration. The plots of the normalized sample weight against temperature for both P(EGDA) and TiO_2_–P(EGDA) beads are shown in Figure 5. The weight changes of P(EGDA) as a function of temperature are represented by the black line in Figure 5. A small weight loss in the region from 20 to 150 °C was due to water evaporation from the beads. When temperature was further increased, more significant weight loss was recorded due to chemical degradation of the sample. The maximum rate of weight loss was observed in the temperature range of 320–380 °C which can be attributed to the degradation of functionalities present in the polymeric network of P(EGDA) and degradation of the main backbone of polymeric chains. The weight changes of TiO_2_–P(EGDA) beads are shown by the red line in Figure 5. A slight weight loss in the range 20–200 °C was due to removal of water from the beads. Interestingly, TiO_2_–P(EGDA) hybrid beads started to degrade at a higher temperature than pure P(EGDA) beads, and the maximum rate of weight loss due to degradation of polymeric network was also observed at higher temperatures (350–400 °C). Therefore, TiO_2_–P(EGDA) hybrid beads exhibit higher thermal stability than pure P(EGDA) beads, probably due to the higher thermal stability of TiO_2_ NPs compared to P(EGDA). The residual weight was nearly the same for both samples due to the small loading of NPs in the polymer matrix. The improved thermal stability of hybrid beads compared to pure polymer beads is in agreement with previously reported TGA results for TiO_2_-polymer nanocomposites^30,31^ and could be a useful feature in the case of thermal regeneration of the beads. The presence of TiO_2_ NPs in P(EGDA) beads not only improves their thermal stability but also imparts photocatalytic activity.

**Figure 6:**
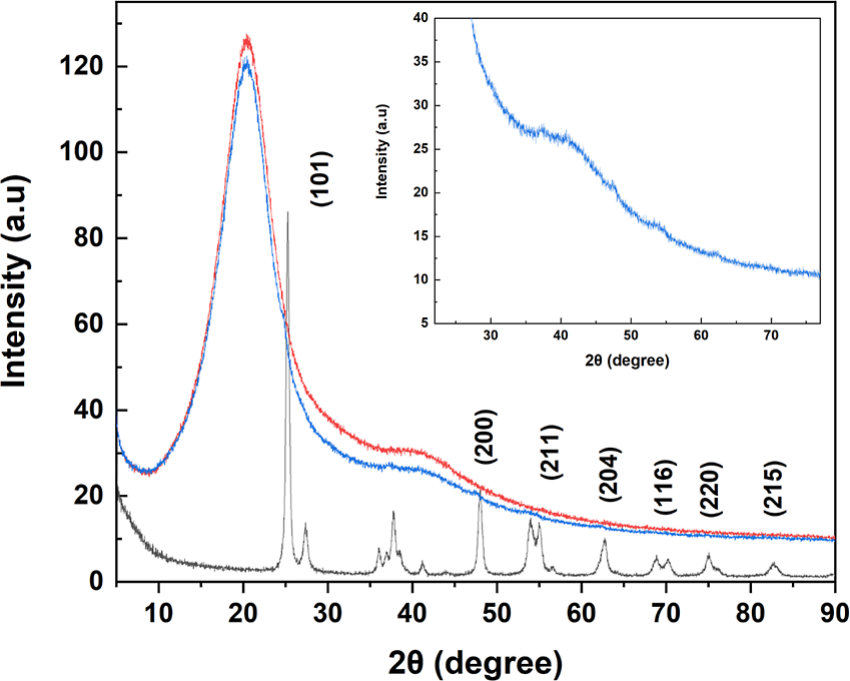
XRD analysis of TiO_2_ (gray line), P(EGDA) beads (red line), and TiO_2_–P(EGDA) hybrid beads (blue line). Inset shows the XRD analysis of TiO_2_–P(EGDA) hybrid beads for clarity of the peaks.

The XRD analysis was performed to confirm the entrapment of TiO_2_ NPs within P(EGDA) beads (Figure 6). Two broad peaks at 2θ values of 20 and 42.5° were related to P(EGDA). These broad peaks are common in the XRD pattern of both pure and hybrid beads, revealing that P(EGDA) has an amorphous structure in both samples. In the XRD pattern of TiO_2_–P(EGDA) beads, the small peaks at 2θ values of 25.3, 37.7, 49.2, 55.4, 62.9, 69.4, 70.6, and 75.6° correspond to the (101), (200), (211), (204), (116), (220), and (215) planes of TiO_2_, which is a clear indication of successful loading of TiO_2_ NPs into P(EGDA).^32^ This result was further confirmed by recording an XRD pattern of TiO_2_ NPs (gray line in Figure 6). Both samples (TiO_2_ NPs and TiO_2_–P(EGDA) beads) have peaks at the same 2θ values. These sharp peaks were missing in the XRD pattern of the pure beads (red line).

### Adsorption of MB on TiO_2_–P(EGDA) Hybrid Beads

The adsorption of MB onto TiO_2_–P(EGDA) beads occurs under the influence of van der Waals forces and hydrogen bonding. The adsorption equilibrium was studied by adding 55 mg of the beads into 30 mL of MB solution at 21 °C and pH 7. The initial dye concentration was 0.25, 0.5, 1, 2.5, 5, and 7.5 ppm and the suspension was stirred in the dark until the adsorption equilibrium had been established. The equilibrium MB concentration in the liquid phase was measured based on the height of the absorbance peak at 664 nm.

**Figure 7:**
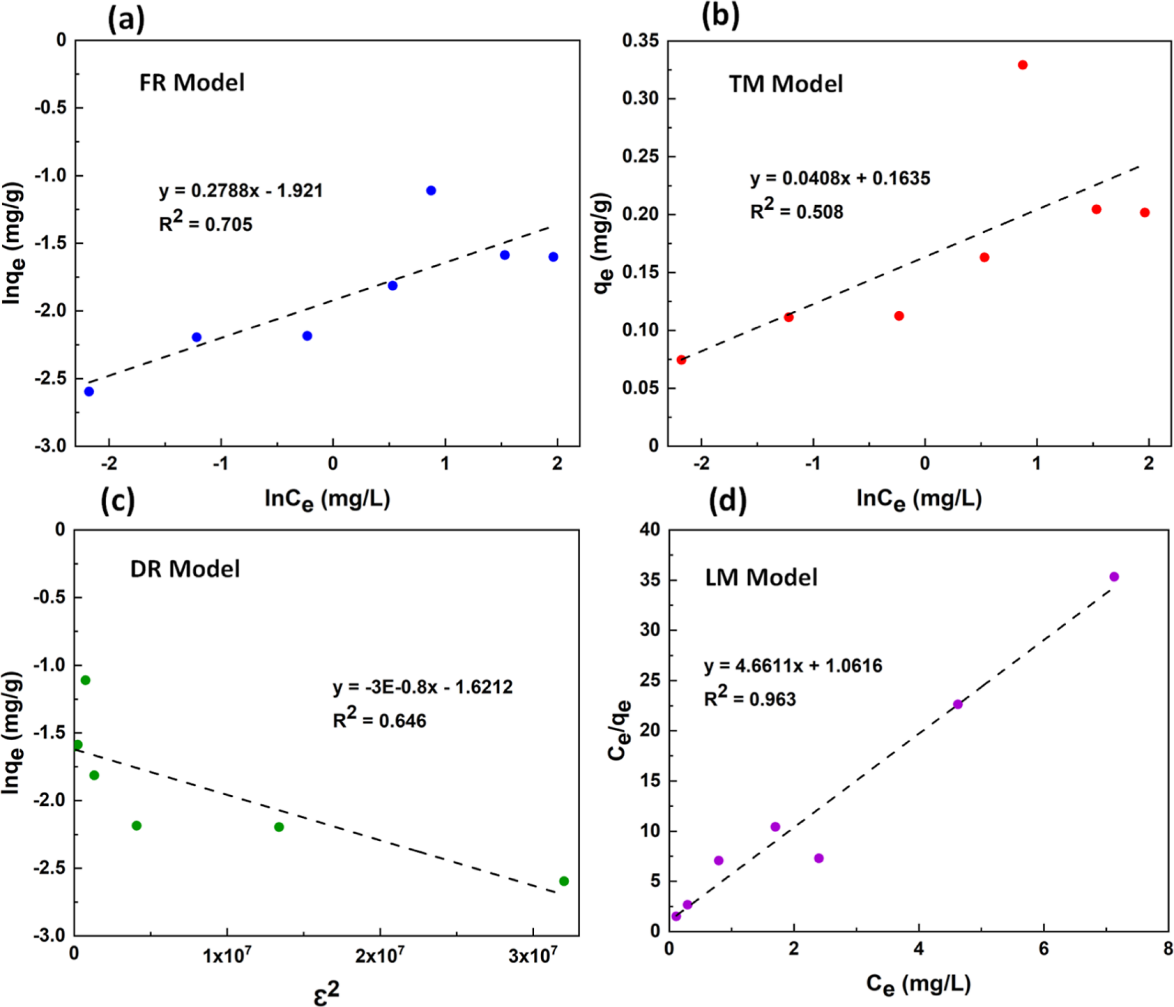
Plots of (a) Freundlich (FR); (b) Temkin (TM); (c) Dubinin–Radushkevich (DR); and (d) Langmuir (LM) isotherm models for adsorption of MB on TiO_2_–P(EGDA) hybrid beads.

Four different adsorption isotherm models, Freundlich (FR), Temkin (TM), Dubinin–Radushkevich (DR), and Langmuir (LM), were used to correlate the mass of MB adsorbed per unit mass of beads (*q*_e_) and the equilibrium concentration of MB in the liquid phase (*C*_e_).^33,34^ A linear form of the FR isotherm is given by eq 1
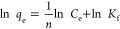
1where *K*_f_ and *n* are Freundlich constants, which can be determined by plotting ln *q*_e_ vs ln *C*_e_, as shown in Figure 7a. The values of *K*_f_ and *n* were calculated from the intercept and slope of the line of best fit, respectively (Table 1). However, since the value of the regression coefficient (*R*^2^ = 0.705) is very low, the adsorption of MB on TiO_2_–P(EGDA) microgel does not follow the FR model.

**Table 1:** Adsorption Parameters for MB Adsorption on TiO_2_–P(EGDA) Hybrid Beads Were Calculated from Langmuir, Freundlich, Dubinin–Radushkevich, and Temkin Models

Langmuir (LM) model				Freundlich (FR) model		
*q*_m_ (mg/g)	*B*	*R*_L_	*R*^2^	*n*	*K*_f_ (L/g)	*R*^2^
0.215	4.38	0.084	0.963	3.6	6.83	0.705
Dubinin–Radushkevich (DR) model				Temkin (TM) model		
*q*_DR_ (mg/g)	β (mol^2^/kJ^2^)	*E* (kJ/mol)	*R*^2^	*B*_T_ (J/mol)	*K*_T_ (L/g)	*R*^2^
5.06	3 × 10^–^^8^	6.39 × 10^3^	0.646	0.1635	0.0408	0.508

A linear form of the TM adsorption model is given by eq 2
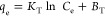
2where *B*_T_ and *K*_T_ are TM constants, which can be determined by plotting *q*_e_ vs ln *C*_e_, as shown in Figure 7b. The values of *B*_T_ and *K*_L_ found from the intercept and slope of the best-fit line are shown in Table 1. However, since *R*^2^ is only 0.508, the TM adsorption model is not applicable here.

A linear form of the DR adsorption isotherm is given by eq 3
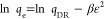
3where *q*_DR_ (mg/g) is the theoretical saturation capacity of MB on the adsorbent surface, while ε and β (mol^2^/kJ^2^) are DR constants. The value of ε was calculated by using the expression, ε = *RT* ln[1 + 1/*C*_e_], where *R* is the universal gas constant and *T* is the absolute temperature. The values of *q*_DR_ and β were determined from the slope and intercept of the ln *q*_e_ vs ε^2^ graph, as shown in Figure 7c, while the mean free energy per molecule was calculated from the equation, *E* = [1/(2β)^1/2^]. The obtained values of *q*_DR_, β, and *E* are given in Table 1. Since *R*^2^ is 0.6458, the adsorption of MB on TiO_2_–P(EGDA) does not follow DR adsorption isotherm modeling, which means that the adsorption of MB onto the hybrid microgel particles is based on layer-by-layer surface coverage rather than pore filling.

The LM adsorption model considers monolayer adsorption with no further adsorption after formation of the monolayer because an equilibrium is established. A linear form of the LM model is given by eq 4
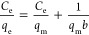
4where *q*_m_ is the maximum adsorption capacity of hybrid beads. The values of *q*_m_ and *b* were determined from the slope and intercept of the *C*_e_/*q*_e_ vs *C*_e_ plot shown in Figure 7d and shown in Table 1. A high *R*^2^ value (0.9634) suggests that the adsorption of MB on a hybrid microgel is following the LM adsorption mechanism. Furthermore, the separation factor (*R*_L_) was calculated from the expression, *R*_L_ = 1/(1 + *bC*_0_), where *C*_0_ is the initial dye concentration in the solution. The *R*_L_ value is used to check the favorability of the adsorption process.^35,36^ The favorable adsorption is characterized by the value of *R*_L_ in the range of 0–1.^37,38^ The *R*_L_ value for the adsorption of MB on TiO_2_–P(EGDA) beads was found to be 0.084, which confirms a favorable adsorption process in this case. It can be concluded that LM is the best fitted model to describe the adsorption of MB on the TiO_2_–P(EGDA) hybrid beads. It agrees with previous adsorption studies performed using various organic dyes and polymer composites.^39−41^

### Kinetics Models for Adsorption of MB on TiO_2_–P(EGDA) Hybrid Beads

**Figure 8:**
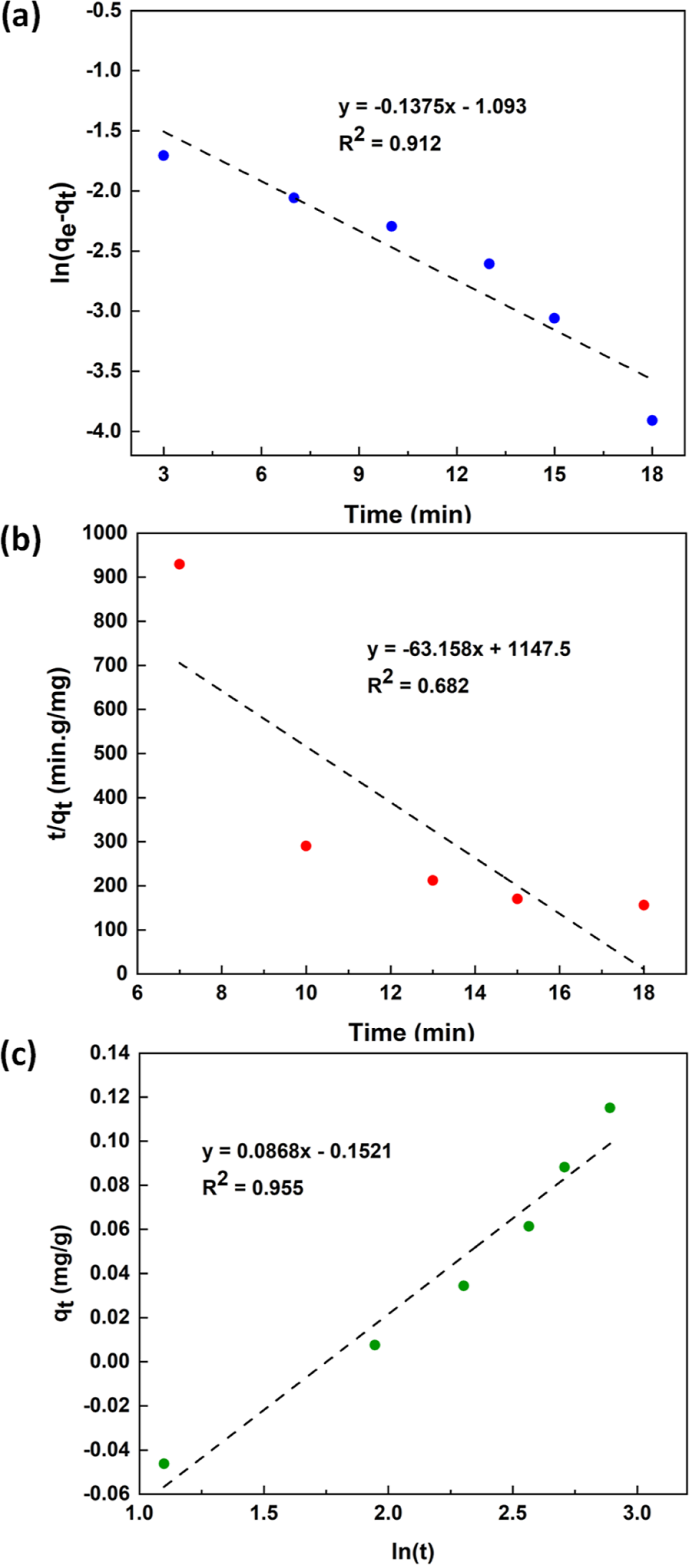
Fitting various kinetic models to the experimental data for the adsorption of MB upon the surface of TiO_2_–P(EGDA) beads. (a) Pseudo-first-order model; (b) Pseudo-second-order model; (c) Elovich model. In each case, 55 mg of the beads were added into 30 mL of a stirred MB solution (1 ppm).

The kinetics of adsorption of MB on TiO_2_–P(EGDA) microgel has been studied using various kinetic models, such as pseudo-first-order, pseudo-second-order, and Elovich models.^42,43^ A linear form of the pseudo-first-order and pseudo-second-order kinetic models is given by eqs 5 and 6, respectively
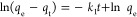
5
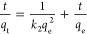
6where *q*_e_ and *q*_t_ (mg/g) are the mass of MB adsorbed per unit mass of microgel beads at equilibrium and at any time, respectively, *k*_1_ (min^–1^) is the pseudo-first-order rate constant, and *k*_2_ (g·mg^–1^·min^–1^) is the second-order rate constant. Equation 5 was tested in Figure 8a by plotting ln(*q*_e_ – *q*_t_) as a function of time, *t*. The values of *k*_1_ and *q*_e_ determined from the slope and intercept of the graph are given in Table 2.

**Table 2:** Kinetic Parameters Determined from the Pseudo-First-Order, Pseudo-Second-Order, and Elovich Models for the Adsorption of MB onto TiO_2_–P(EGDA) Hybrid Beads^a^

Pseudo-first-order kinetic parameters		
*k*_1_ (min^–1^)	*q*_e_ (mg·g^–^^1^)	*R*^2^ (—)
0.132	2.98	0.912
Pseudo-second-order kinetic parameters		
*k*_2_ (g·mg^–^^1^·min^–^^1^)	*q*_e_ (mg·g^–^^1^)	*R*^2^ (/)
3.5	0.0158	0.682
Elovich model parameters		
α (mg·g^–^^1^·min^–^^1^)	β (g·mg^–^^1^)	*R*^2^ (—)
0.0881	11.5	0.955

a The coefficients of determination, *R*^2^, are also provided to estimate the goodness of fit in each case.

In Figure 8b, *t*/*q*_t_ was plotted against time, *t* using eq 6. The values of *k*_2_ and *q*_e_ determined from the slope and intercept of the line are given in Table 2. The *R*^2^ values for fitting the pseudo-first and pseudo-second order rate model are 0.912 and 0.682, respectively, confirming that the pseudo-first-order kinetic model is more accurate for the adsorption of MB on the hybrid beads. This finding is in agreement with the behavior observed in earlier studies for the adsorption of MB on polyaniline/graphene oxide or polyaniline/reduced graphene oxide composites.^44^

Moreover, the Elovich chemisorption model was also employed
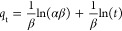
7where α and β are the initial adsorption rate constant and the desorption constant, respectively. The fitting of the experimental data to the Elovich kinetic model is shown in Figure 8c. The values of α and β determined from the intercept and slope of the *q*_t_ vs ln(*t*) given in Figure 8c are shown in Table 2. Based on the *R*^2^ values, the Elovich model can be considered as the most accurate model for the prediction of the adsorption kinetics of MB on the TiO_2_–P(EGDA) microgel.

### Photocatalytic Activity of TiO_2_–P(EGDA) Hybrid Beads

**Figure 9:**
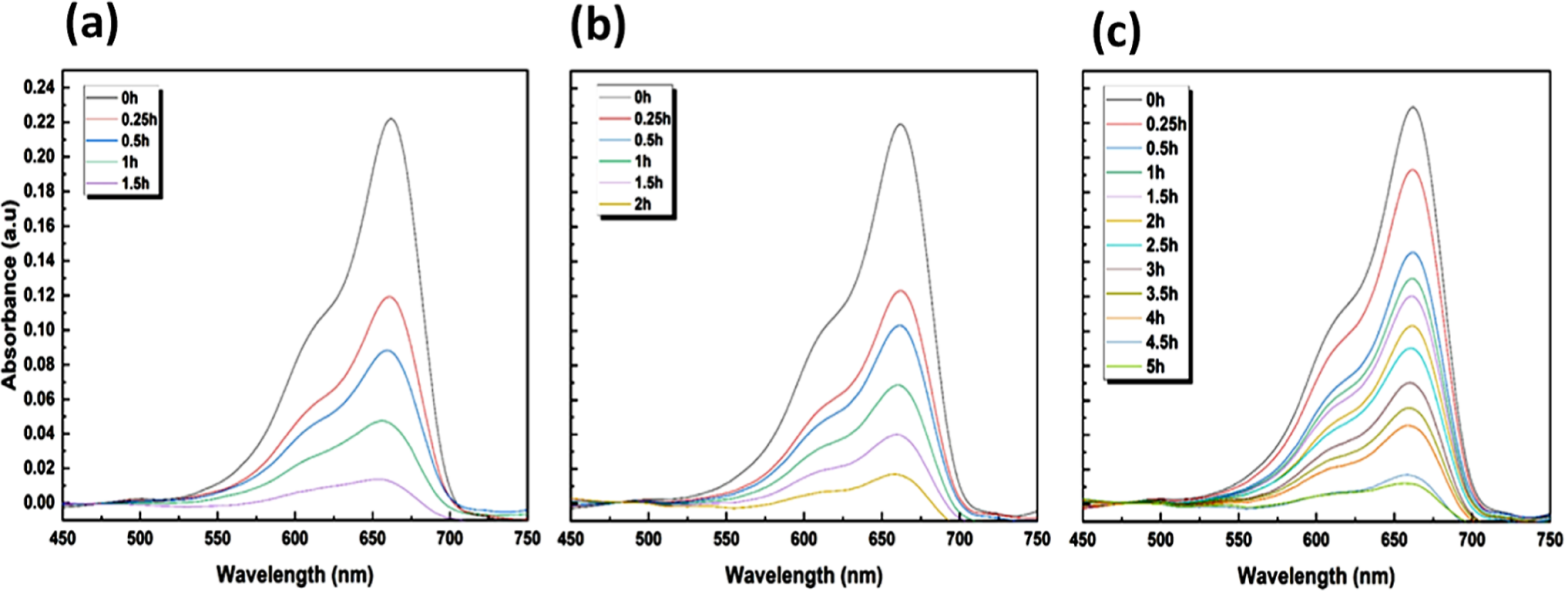
UV–visible absorption spectra of MB solution as a function of time in the presence of TiO_2_–P(EGDA) (CB) hybrid beads at (a) 8; (b) 4; and (c) 2 W/m^2^ UV light irradiance. In each experiment, 0.13 g/L TiO_2_–P(EGDA) beads were added into 20 mL of well-stirred MB solution (1 ppm).

Dyes are important industrial chemicals, which are disposed of in large quantities into water bodies from various industries like textiles, paint, leather, food, and pharmaceuticals.^45,46^ They can be removed from water by chemical oxidation, adsorption, and photocatalysis. Photocatalysis holds several advantages over competing technologies, including complete and fast dye removal without causing secondary pollution, no need for additional chemical agents, and low operating costs since the process can be driven by solar energy.^47^ The added benefits of using hybrid TiO_2_-filled hydrogel beads for photocatalysis are their tunable size, biocompatibility, and excellent recyclability, due to the large size of microbeads compared to the size of TiO_2_ NPs. In this study, the photocatalytic degradation of MB was chosen as a model reaction to demonstrate the photocatalytic activity of the fabricated beads. For this purpose, 20 mL of a 1 ppm MB solution containing 0.13 g/L of TiO_2_–P(EGDA) (CB) was exposed to UV light at 2, 4, and 8 W/m^2^ irradiances, and the progress of dye degradation was monitored by spectrophotometric analysis, as shown in Figure 9. During photocatalytic degradation, the central heterocyclic ring of MB is broken and sulfhydryl (−S^+^=) group is oxidized by hydroxyl radicals (^•^OH) to produce a sulfonyl group.^48^ A steady decrease in the height of the absorption peak at 664 nm was observed at all light intensities, since the absorption wavelength of the sulfonyl group is less than 180 nm. This result shows that TiO_2_–P(EGDA) (CB) hybrid beads are photocatalytically active, even at very low UV light irradiance of 2 W/m^2^.

As can be seen from Figure 9, the UV light irradiance had a large impact on the rate of degradation of MB. When the solution was irradiated with 8 W/m^2^, the peak height was reduced from 0.22 to 0.02 in 1.5 h, as shown in Figure 9a. At 4 W/m^2^, the peak height was reduced to the same level in 2 h, as reflected in Figure 9b. When the UV light irradiance was 2 W/m^2^, it took 5 h for the peak height to be reduced to 0.02, Figure 9c. A strong correlation between the reaction time and the UV irradiance confirms that the decrease in absorbance was due to a photocatalytic reaction and not due to physical adsorption or catalysis. The degradation efficiency of MB is generally smaller when less UV radiation is emitted from the sample since less reactive oxygen species are produced. At the low UV irradiances used in this study, the rate of photocatalytic degradation is usually proportional to the irradiance level.^49^ At high UV irradiances (>25 W/m^2^), the dye degradation rate is expected to be independent of the intensity of UV light since a dynamic equilibrium is reached between the reactions leading to electron–hole pair formation and electron–hole recombination.

**Figure 10:**
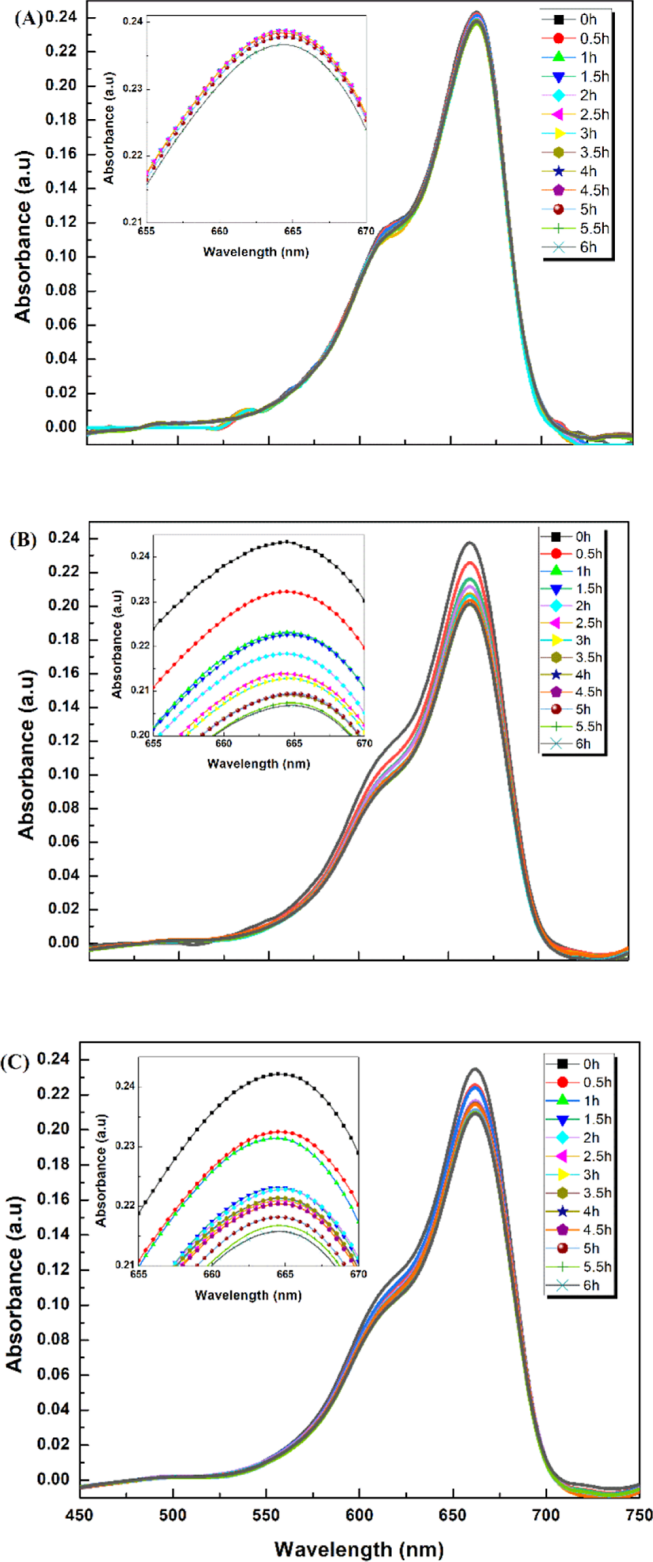
UV–visible absorption spectra of 1.0 ppm of MB solution as a function of time (a) under UV light (8 W/m^2^) but without photocatalyst; (b) in the presence of TiO_2_–P(EGDA) catalytic beads (25 g/L) (CB) in the dark; and (c) in the presence of P(EGDA) beads (25 g/L) under UV light (8 W/m^2^).

Three control experiments were performed to confirm that both TiO_2_–P(EGDA) (CB) beads and UV light are needed for the rapid degradation of MB (Figure 10). In the first experiment, 1 ppm of MB solution was irradiated with 8 W/m^2^ UV light without adding TiO_2_–P(EGDA) beads. No decrease in absorption peak was observed within 6 h, confirming that UV light cannot degrade MB without hybrid beads, as given in Figure 10a. In the next experiment, hybrid beads were added to 1 ppm MB solution, and the reaction mixture was stirred in the dark. No prominent decrease of absorbance peak was observed, as shown in Figure 10b, and a small decline in absorbance from 0.24 to 0.20 after 6 h can be attributed to the dye adsorption on the beads surface. When the same MB solution was exposed to UV light at the same irradiance (8 W/m^2^) and catalyst loading (25 g/L), the absorbance decreased from 0.22 to 0.02 in 1.5 h, Figure 9a. Therefore, both hybrid beads and UV light are needed for the photocatalytic degradation of MB.

In the third experiment, P(EGDA) beads were added to 1 ppm of MB solution, and the dye removal was monitored over 6 h under the UV light irradiation of 8 W/m^2^. As shown in Figure 10c, again a very small decrease in the absorbance peak occurred, which can be attributed to the physical adsorption of MB to P(EGDA) beads. Interestingly, the absorbance decreased by a smaller amount in the presence of pure polymer beads than TiO_2_–P(EGDA) (CB) beads, meaning that the dye adsorption was enhanced by entrapped TiO_2_ NPs. This experiment confirmed that P(EGDA) microgel particles cannot serve as photocatalysts for the degradation of MB, unless TiO_2_ NPs are entrapped into the P(EGDA) matrix.

**Figure 11:**
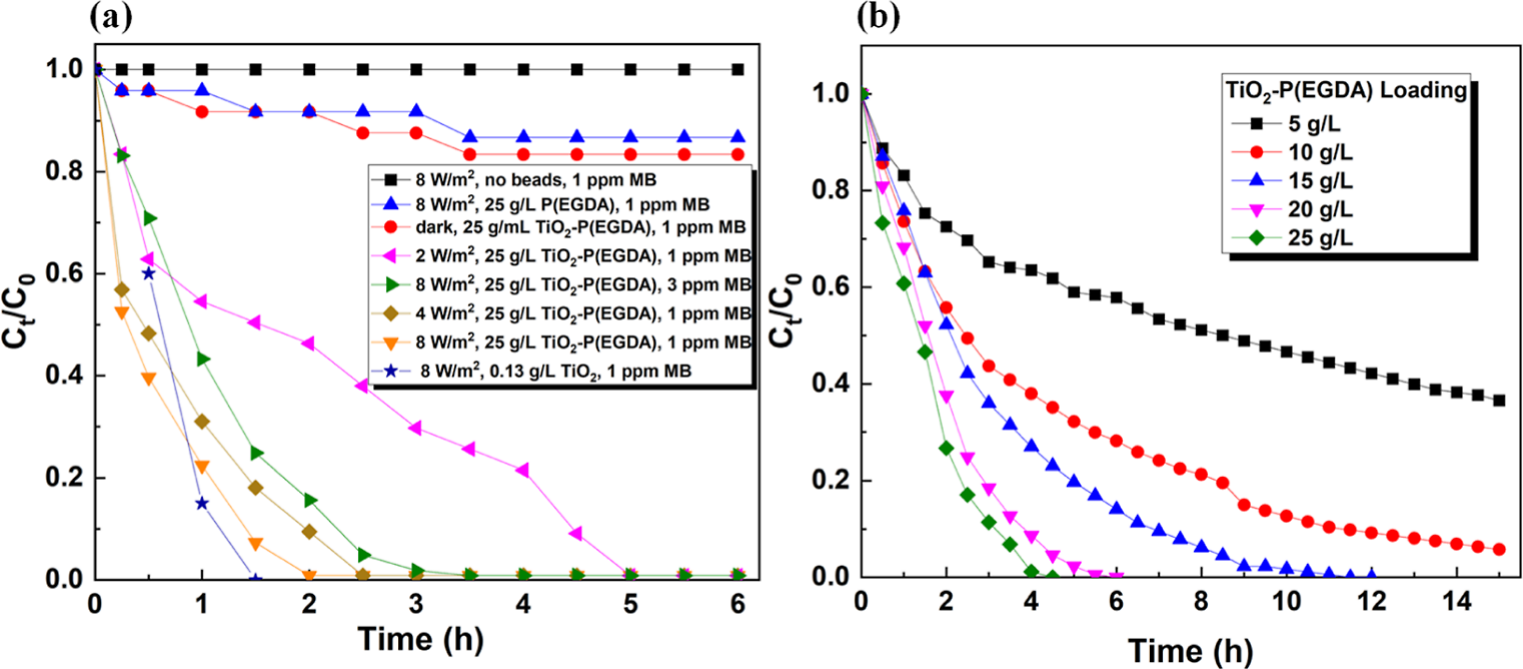
(a) *C*_t_/*C*_0_ vs time curves for photocatalytic degradation of 1 and 3 ppm MB solution under different reaction conditions; (b) *C*_t_/*C*_0_ vs time curves for photocatalytic degradation of 10 ppm MB solution under 8 W/m^2^ UV irradiation at different loadings of TiO_2_–P(EGDA) (CB) hybrid beads.

The reaction progress was also monitored by plotting *C*_t_/*C*_0_ against time for the experiments carried out at different light intensities, initial concentrations of MB, and catalyst loadings, Figure 11a,b. As a comparison, the results of three control experiments are also plotted in Figure 11a.

The *C*_t_/*C*_0_ vs time plots for the degradation of MB from 1 and 3 ppm solutions at different UV light irradiances at the constant loading of catalytic beads (25 g/L) are shown in Figure 11a. The results obtained with pure TiO_2_ NPs are also included in this figure. In this case, 0.13 g/L of pure TiO_2_ was added to 1 ppm MB solution and the sample was exposed to UV light at 8 W/m^2^. It should be noted that 0.13 g/L pure TiO_2_ is equivalent to 25 g/L catalytic beads since the content of TiO_2_ in the catalytic beads was 0.5 wt %. After 0.5 h, the catalytic beads were more efficient than pure TiO_2_ and degraded 61% of MB, while pure TiO_2_ NPs removed only 40% of the dye. However, pure TiO_2_ NPs were more efficient over longer times and degraded 85% of MB after 1 h and 100% of MB after 1.5 h. On the other hand, catalytic beads degraded 78% of MB after 1 h, 93% after 1.5 h, and 100% after 2 h. A relatively small difference in performance between pure TiO_2_ and TiO_2_-loaded beads shows that the catalytic beads exhibit small internal diffusional resistances and high transmittance of UV light.

Except for irradiation at 2 W/m^2^, the MB degradation efficiency was almost 100% after 4 h of irradiation. Rauf et al.^50^ have achieved degradation efficiency of MB of 70% from 1.6 ppm solution after 4 h of irradiation using 0.64 g/L photocatalyst (Cr–Ti binary oxide with 10 mol % Cr^3+^). Although the TiO_2_ loading in this study was much smaller (0.025 g/L), the dye degradation efficiency was significantly higher.

By increasing the UV light irradiance, the degradation rate of MB increases since more electron–hole pairs are generated in TiO_2_ NPs. The *C*_t_/*C*_0_ vs time graph for photocatalytic degradation of a 10 ppm MB solution using different catalyst loadings is given in Figure 11b. The rate of photocatalytic degradation increases by increasing the amount of TiO_2_–P(EGDA) beads added to the reaction mixture, and the reaction can be completed faster. The Langmuir–Hinshelwood (LH) kinetic model is widely accepted to describe the kinetics of photocatalytic degradation of MB.^1^ The LH mechanism involves the coadsorption of MB and H_2_O molecules on the catalyst surface, followed by a surface reaction between the adsorbed water molecules and positive holes in the valence band, leading to the generation of hydroxyl radicals. The dissolved oxygen can also be adsorbed on the catalyst surface and react with electrons in the conduction band to form a superoxide anion (O_2_^•–^). The formation of O_2_^•–^ triggers a series of reactions leading to the formation of various other reactive oxygen species, such as hydrogen peroxide (H_2_O_2_), hydroperoxyl radical (HO_2_^•–^), and hydroxyl radical (^•^OH).^51^ Finally, the released reactive oxygen species react with the adsorbed MB molecules, causing their breakdown into smaller molecules.^48^ According to the LH model, the greater the catalyst surface area, the more adsorption sites there are and the faster the reaction, as shown in Figure 11b.

Initially, the reaction rate strongly depends on the catalyst loading since the rate-limiting factor at low catalyst loadings is the availability of active sites on the catalyst surface. As the catalyst loading increases, this effect weakens and the rate-limiting factor becomes the rate of surface reaction, as can be seen by the small difference in the reaction rate when 20 and 25 g/L beads were added. Further increase in catalyst loading might have a negative impact on the reaction rate due to light scattering by the beads, leading to poor light utilization.^52^ Without TiO_2_ NPs in the polymer matrix or without UV light, the removal efficiency of MB was only 13 and 16%, respectively, as compared to ∼100% in the presence of photocatalytic beads and UV light, Figure 11b. Also, when no particles were added to the reaction mixture, the removal efficiency was zero, irrespective of the irradiation time and UV light irradiance.

The reaction kinetics was investigated using the pseudo-first-order model based on eq 8
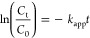
8

**Figure 12:**
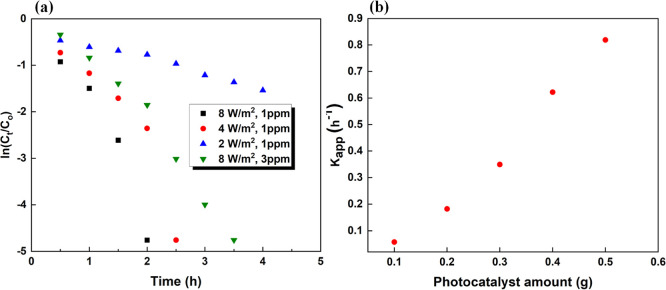
(a) Plots of ln(*C*_t_/*C*_0_) vs time for the photocatalytic degradation of MB solution of different initial concentrations under different UV light intensities; (b) apparent rate constant (*k*_app_) for photocatalytic degradation of 10 ppm MB solution under 8 W/m^2^ UV irradiation in the presence of different amounts of TiO_2_–P(EGDA) catalytic beads.

A linear portion of the ln(*C*_t_/*C*_0_) vs time plots shown in Figure 12a was used to calculate the apparent rate constant (*k*_app_) for photocatalytic degradation of MB under different conditions, and the obtained *k*_app_ values are listed in Table 3.

**Table 3:** Apparent Rate Constant (*k*_app_) for the Photocatalytic Degradation of MB at Different Loadings of Catalytic Beads in the Dye Solution, UV Light Irradiances, and Initial Dye Concentrations

UV light irradiance (W/m^2^)	mass of catalytic beads (g)	concentration of beads (g/L)	initial MB concentration (ppm)	*k*_app_ (h^–1^)
8	0.1	5	10	0.057
8	0.2	10	10	0.182
8	0.3	15	10	0.349
8	0.4	20	10	0.622
8	0.5	25	10	0.819
2	0.5	25	1	0.311
4	0.5	25	1	1.85
8	0.5	25	1	2.52

As shown in Figure 12b, the apparent rate constant increased linearly with increasing catalyst loading, reflecting the fact that the number of active adsorption sites on the catalyst surface is a linear function of the catalyst loading. Also, at constant UV light irradiance (8 W/m^2^) and the beads loading in the solution (25 g/L), *k*_app_ decreased from 2.52 to 0.819 h^–1^ when the initial MB concentration increased from 1 to 10 ppm (Table 3). Due to increased adsorption of MB molecules on the catalyst surface at the higher dye concentration, a smaller fraction of active sites was available for the adsorption of water molecules, and a smaller number of ^•^OH radicals were produced.^52^ Also, as the dye concentration increases, more and more UV light is absorbed by the dye molecules dissolved in the liquid phase and adsorbed onto the catalyst surface, which can prevent photons from reaching the surface of TiO_2_ NPs.^53^

### Comparison of Photocatalytic Activity of TiO_2_–P(EGDA) with Previous Studies

Usually, TiO_2_ NPs are coated on the surface of a support (polystyrene,^54^ Si@Fe,^55^ reduced graphene oxide)^56^ for photocatalytic degradation of MB due to which the photocatalytic system does not remain stable for their reuse in large number of cycles. Moreover, high light irradiance is needed for the complete degradation of MB. A comparison of photocatalytic activity of TiO_2_–P(EGDA) with work in terms of photocatalytic activity and recyclability (removal efficiency at different cycles) reported in the literature is presented in Table 4.

**Table 4:** Comparison of Different Systems Containing TiO_2_ NPs for the Photocatalytic Degradation of MB

catalyst	light irradiance (W/m^2^)	MB concentration (ppm)	dosage (g/L)	*k* (h^–1^)	recyclability	refs
PS(TiO_2_) core–shell	1500	7	10	216	not reported	(54)
TiO_2_-(Si@Fe) core–shell	12,700	10	0.15	0.66	40% at 5th cycle	(55)
rGo/TiO_2_	250	60	0.4	3.93	86.24% at 10th cycle	(56)
TiO_2_–P(EGDA)	8.00	10	25	0.819	100% at 10th cycle	this work

**Figure 13:**
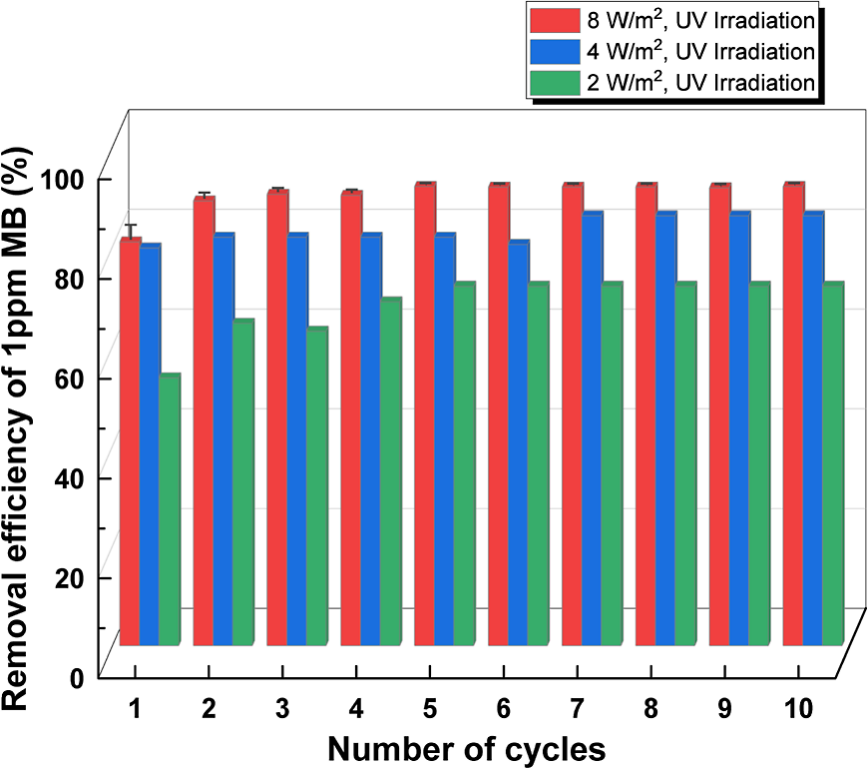
Efficiency of photocatalytic degradation of MB from 1 ppm solution in 10 consecutive runs using recycled TiO_2_–P(EGDA) catalytic beads (CB). The reaction time in each cycle was 1.5 h, and the concentration of CBs in the suspension was 25 g/L (the content of TiO_2_ NPs in the suspension was 0.025 g/L).

A catalyst recyclability study was also performed by repeating degradation experiments with 1 ppm of MB solution 10 times under 8, 4, and 2 W/m^2^ UV irradiance using the same photocatalyst. After each cycle, the photocatalyst was separated from the reaction mixture by gravity settling, which took only 10 s. After separation, the photocatalytic beads were regenerated by washing 5–7 times with pure acetone, followed by 3–5 washes with deionized water. The highest removal efficiency of MB (nearly 100%) was achieved at the highest irradiance level (8 W/m^2^). It should be noted that the removal efficiency at smaller UV irradiances would eventually reach 100% if the time of UV exposure in each cycle was longer. Moreover, no loss in photocatalytic efficiency of TiO_2_–P(EGDA) (CB) beads was observed after 10 cycles, indicating that TiO_2_ NPs were neither released from the polymer matrix nor deactivated. Furthermore, a slight increase in photocatalytic activity occurred in the first several cycles, probably due to complete removal of surfactant molecules from the catalyst surface. As shown in Figure 4, the silicon surfactant was not fully removed from the particle surfaces during washing. Therefore, some active sites on the catalyst surface were covered by surfactant molecules and unavailable to the reactant molecules. During UV irradiation, the surfactant molecules were degraded by the photocatalyst just like the dye and, consequently, the removal efficiency was higher in the subsequent cycles. At the smaller light intensities, more cycles were needed to fully remove the surfactant molecules from the interface. A steady-state removal efficiency of MB under the experimental conditions shown in Figure 13 varied from 72% at 2 W/m^2^ to 92% at 8 W/m^2^.

## Conclusions

Nearly monodisperse P(EGDA) microgel beads and TiO_2_–P(EGDA) hybrid beads were successfully prepared by 3D hydrodynamic flow focusing using a Lego-inspired glass capillary microfluidic device. The synthesized hybrid microgel beads were perfectly spherical in shape, with a smooth surface, and dot-like protrusions from embedded TiO_2_ NPs. The hybrid microgel beads showed excellent photocatalytic activity for the removal of a trace amount of methylene blue (1–10 ppm) from aqueous solutions. The adsorption data were found to be best fitted with the Langmuir adsorption isotherm. P(EGDA) not only stabilizes TiO_2_ NPs but also enhances their recyclability and photocatalytic ability. The mesh size of the P(EGDA) polymer network was large enough to allow diffusion of MB molecules to the surface of embedded TiO_2_ NPs, which allowed for the dye removal efficiency of nearly 100% from 1 and 3 ppm solutions. Photocatalytic activity of the hybrid system could be controlled by varying the reaction conditions, such as UV light irradiance and the amount of TiO_2_–P(EGDA) beads in the reaction mixture. The photocatalytic microgel beads could be recycled and used in multiple cycles without any loss of their photocatalytic efficiency. The developed hybrid beads can be used in the future for photocatalytic degradation of other dyes and other persistent organic pollutants. The microfluidic device can be cheaply fabricated by computer numerical control (CNC) milling and is easy to assemble, operate, and dismantle for cleaning. The size of the microgel beads can be precisely controlled by hydrodynamic conditions and flow geometry in the microfluidic device, which allows for precise tuning of the photocatalytic performance of the beads and their behavior in the reactor, such as settling time and flow properties.
